# Case report: Identification of three novel compound heterozygous SGLT2 variants in three Chinese pediatric patients with familial renal glucosuria

**DOI:** 10.3389/fped.2022.996946

**Published:** 2022-11-28

**Authors:** Huimei Huang, Xiantao Wu, Qing He, Xuqin Liang, Yi Ding, Zhijuan Li, Zhanping Ren, Ying Bao

**Affiliations:** ^1^ Department of Nephrology, Xi'an Children's Hospital, The Affiliated Children's Hospital of Xi'an Jiaotong University, Xi’an, China; ^2^ Department of Physiology and Pathophysiology, School of Basic Medical Sciences, Xi'an Jiaotong University, Xi’an, China; ^3^ Key Laboratory of Shaanxi Province for Craniofacial Precision Medicine Research, College of Stomatology, Xi'an Jiaotong University, Xi’an, China; ^4^ Department of Cleft Lip and Palate Surgery, College of Stomatology, Xi'an Jiaotong University, Xi’an, China

**Keywords:** familial renal glucosuria, SGLT2, genetic kidney disease, whole exome sequencing, gene mutation

## Abstract

Familial renal glucosuria (FRG) is a rare genetic condition featured by isolated glucosuria without hyperglycemia or other kidney diseases. It is caused by pathogenic mutations of the SGLT2 (Sodium-Glucose Cotransporter 2) gene, whose protein product is responsible for reabsorbing the majority of glucose in the early proximal convoluted tubule. Hitherto, quite an array of variants of SGLT2 have been identified in patients of FRG. In this study, we performed whole exome sequencing on three Chinese pediatric patients with FRG and uncovered three compound heterozygous variants of SGLT2: c.1333C > T (p.Q445X) and c.1130–5 C > G; c.1438G > T (p.V480F) and c.346G > A (p.V116M); c.1175C > G (p.S392C) and c.1333C > T (p.Q445X). Among the total of five variants, c.1333C > T (p.Q445X), c.1438G > T (p.V480F) and c.1175C > G (p.S392C) represented novel variants that had not been reported in any genetic databases. All five variants had extremely low allele frequencies and the amino acids loci affected by missense variants were highly conserved in vertebrate species. Bioinformatic tools predicted that all five variants might disrupt the function of SGLT2, which were likely to be causal for FRG in these patients. Our findings expand the variant spectrum of SGLT2 associated with FRG and provide novel insights into mechanism of action of this transporter, which will aid in the development of novel SGLT2 inhibitors for treatment of type 2 diabetes and cardiovascular diseases.

## Introduction

Familial renal glucosuria (FRG) is a rare inherited kidney disease characterized by persistent glucosuria without abnormal serum glucose concentrations and other tubular function perturbations (1). In humans, the majority of glucose is reabsorbed in the early proximal convoluted tubule segment S1 of the kidney by the sodium–glucose cotransporter 2 (SGLT2) which is a 14-transmembrane protein and consists of 672 amino acids (1). Genetic studies have established that pathogenic mutations in SGLT2 are causal for most cases of FRG and increasing numbers of mutations have been being identified with the advance of sequencing technologies (2–9). In the present study, we uncover three compound heterozygous variants of SGLT2 in three Chinese children with FRG and *in silico* analyses suggest that these variants are likely to be pathogenic.

## Methods

### Case presentation

Three pediatric patients from three unrelated families were diagnosed with FRG by the presence of glucosuria and absence of hyperglycemia after urine abnormalities had persisted for 1, 6 and 4.5 months respectively. Clinical characteristics of the patients were described in Supplementary Table S1. Routine urinary analysis showed glucose level above 3+ (28 mmol/L > urine glucose ≥ 14 mmol/L) with no other abnormalities. Since FRG is usually a benign condition and has favorable prognosis, no specific treatment or intervention was implemented to the patients, who were advised to appropriately increase the uptake of carbohydrates and not to overdo exercise. Regular 6-months follow-up was arranged for the patients. Urine analysis showed gradually decreasing glucose level and improving symptoms, indicating a favorable prognosis for the patients. No clinical manifestations, including hypoglycemia, dehydration or ketosis, were observed. No adverse events and accidents occurred. The patients had no history of urinary tract infection. The patients also had no problems with movement, eating, sleeping or excretory function and their body weight gain was normal. There was no reported history of trauma or poisoning. Their parents and other family members had no history of glucosuria.

### Genetic analysis

The peripheral blood of the patients was collected and sent to Fujun Genetics Inc. (Shanghai, China) for whole exome sequencing (WES). Briefly, DNA library was constructed by using the IDT xGen™ Exome Research Panel V2 Kit and then sequenced using an Illumina HiSeq X10 platform using a 100-bp paired-end reads according to the standard procedure. The sequencing data was filtered and aligned with the human reference genome (GRCh38/hg19) by using the BWA Aligner (http://bio-bwa.sourceforge.net/) and variants were annotated by ANNOVAR (annovar.openbioinformatics.org/en/latest/). The candidate causal variants were confirmed by Sanger sequencing, for which the primers were listed in Supplementary Table S2.

## Results

**Figure 1:**
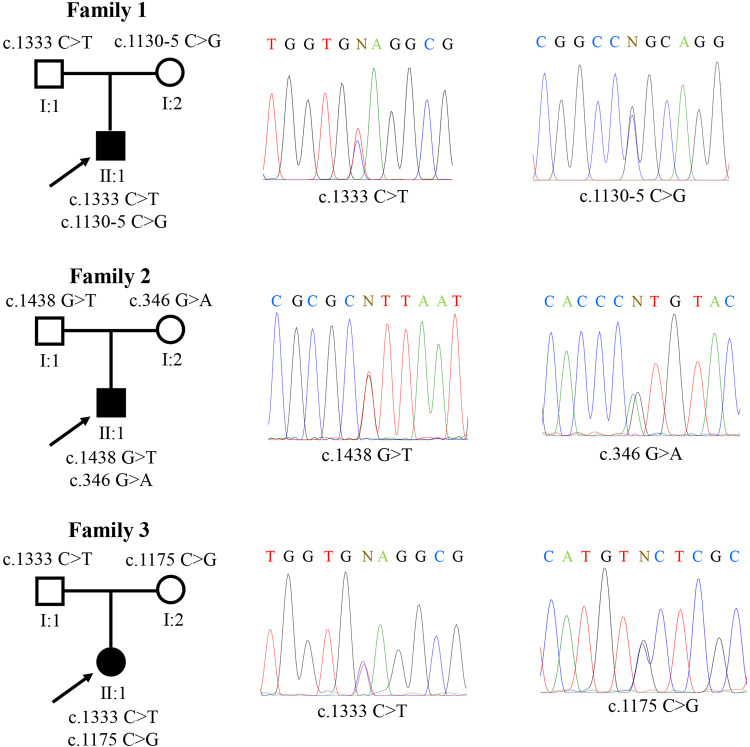
Three familial renal glucosuria pedigrees carrying SGLT2 variants. A total of five different mutations were identified: c.1333C > T (p.Q445X), c.1130-5 C > G, c.1438G > T (p.V480F), c.346G > A (p.V116M) and c.1175C > G (p.S392C). “N” indicates the position of nucleotide substitution.

Three Chinese pediatric probands from three unrelated families, but not their parents, met the diagnostic criteria of FRG (Table 1). Further examination of the patients and their parents revealed no clinical manifestations or any other tubular dysfunctions or other types of kidney diseases. WES was performed on the patients and three compound heterozygous variants were identified in the SGLT2 gene: c.1333C > T (p.Q445X), c.1130–5 C > G, c.1438G > T (p.V480F), c.346G > A (p.V116M) and c.1175C > G (p.S392C). These variants were all confirmed by Sanger sequencing (Figure 1). Notably, for all three compound heterozygous variants, one allele was of maternal origin and the other of paternal origin (Table 1, Figure 1).

**Table 1:** Mutations in SGLT2 and glucose excretion in three Chinese pediatric patients and their parents. In column 1, “age” indicates at the time of evaluation. In column 2, “-” means within the normal range.

Family members (age)	Glucose excretion	Allele 1	Allele 2
**Family 1**			
I:1	–	c.1333 C > T/p.Q445X	WT
I:2	–	WT	c.1130-5 C > G
II:1 (5)	3+	c.1333 C > T/p.Q445X	c.1130-5 C > G
**Family 2**			
I:1	–	c.1438 G > T/p.V480F	WT
I:2	–	WT	c.346 G > A/p.V116M
II:1 (1)	3+	c.1438 G > T/p.V480F	c.346 G > A/p.V116M
**Family 3**			
I:1	–	c.1333 C > T/p.Q445X	WT
I:2	–	WT	c.1175 C > G/p.S392C
II:1 (4)	3+	c.1333 C > T/p.Q445X	c.1175 C > G/p.S392C

To determine the allele frequencies of these variants, we searched different genomic databases. Three of the five variants (p.S392C, p.Q445X, and p.V480F) had not reported among all databases, and thus represented novel variants. Two variants (p.V116M and c.1130–5 C > G) had coding numbers in dbSNP database (rs146835104 for p.V116M and rs760945249 for c.1130–5 C > G). The p.V116M variant was recorded in gnomAD-Exomes (Asian population) with a very low frequency of 0.00002 (3). And the allele frequencies of c.1130–5 C > G was 0 in all searched databases for Asian population (Table 2).

**Table 2:** Allele frequencies for the SGLT2 variants in the Asian population. The version of chromosome database used in this study is GRCh38; the coding sequence positions for the SGLT2 transcript are based on NM_003041; the amino acid positions for the SGLT2 protein are based on NP_003032.

Variants	Type of variants	Position change in chromosome	cDNA change	Amino acid substitution	dbSNP database	East Asian in ALFA	Asian in ExAC	Asian in gnomAD-Exomes	Asian in gnomAD-Genomes
V116M	Missense	chr16:314857751 G > A	c.346 G > A	Val116Met	rs146835104	0	0	0.00002	0
S392C	Missense	chr16:31488667 C > G	c.1175 C > G	Ser392Cys	NR	NR	NR	NR	NR
Q445X	Stop-gain	chr16:31488932 C > T	c.1333 C > T	Gln445Ter	NR	NR	NR	NR	NR
V480F	Missense	chr16:31489037 G > T	c.1438 G > T	Val480Phe	NR	NR	NR	NR	NR
c.1130-5 C > G	Splicing	chr16:31488617: C > G	/	/	rs760945249	0	0	0	NR

“NR” indicates not recorded.

**Figure 2:**
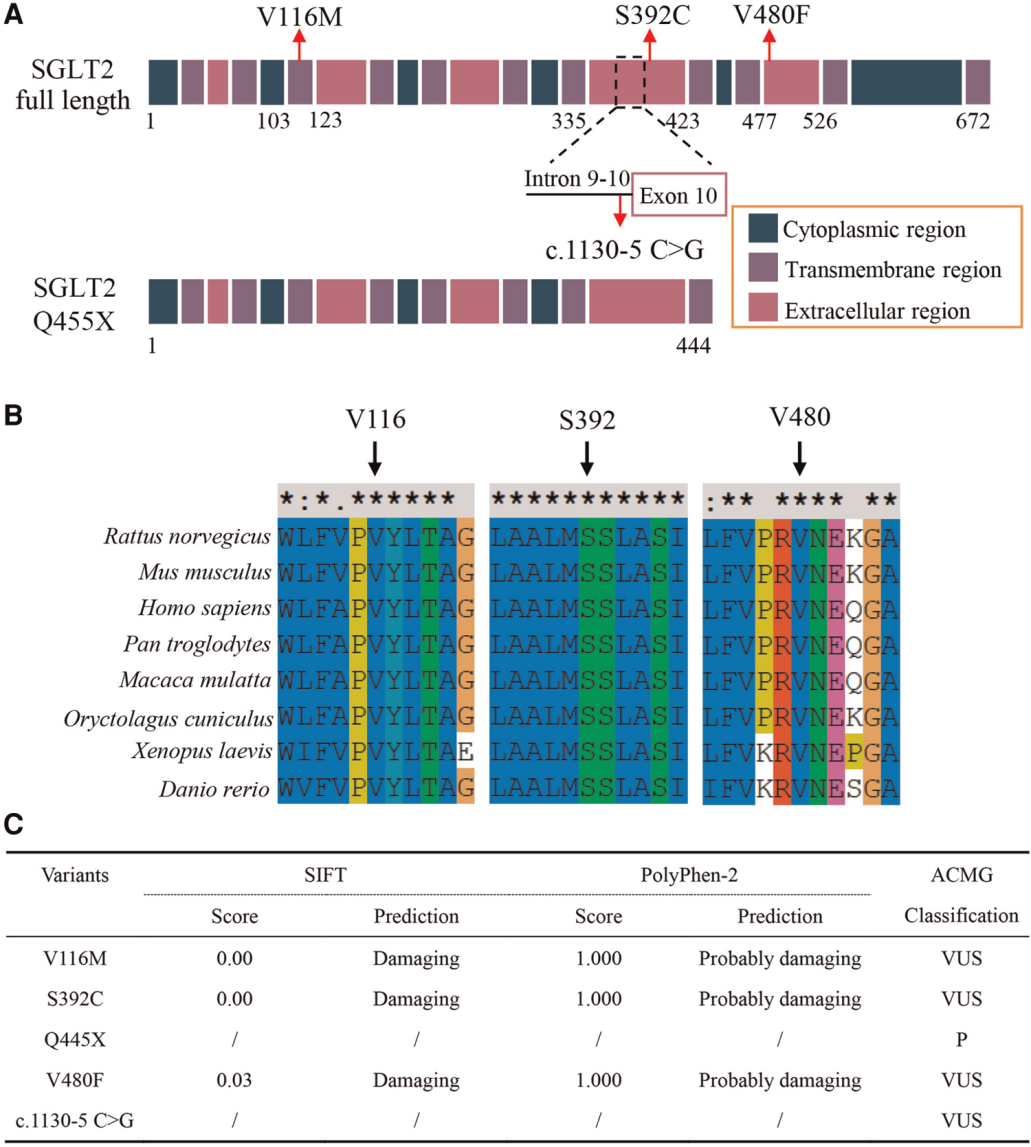
(**A**) Schematic illustration showing SGLT2 protein domains with loci affected by variants indicated. (**B**) Multiple sequence alignment of the SGLT2 protein from different vertebrate species. Arrows indicate amino acids affected by missense variants, which are highly conserved. (**C**) Prediction of the effects of variants on SGLT2 protein function and ACMG classification of the variants. For ACMG classifications: V116M, VUS (PM2 + PP1 + PP3 + PP4); S392C, VUS (PM2 + PP1 + PP3 + PP4); Q455X, P (PVS1 + PM2 + PP1 + PP3 + PP4); V480F (PM2 + PP1 + PP3 + PP4); c.1130-5 C > G, VUS (PM2 + PP1 + PP3 + PP4). VUS, uncertain significance; P, pathogenic. PVS1, null variants (nonsense); PM2, not present (or at extremely low frequency) in normal population; PP1, co-segregation in the families; PP3, predicted to be pathogenic by multiple bioinformatic tools; PP4, family history is highly specific with a single genetic etiology.

We analyzed the pathogenicity of these SGLT2 variants. The p.Q445X was a nonsense variant and caused truncation of the SGLT2 protein, which is usually deleterious. The c.1130–5 C > G variant located in the Intron 9–10, 5 bp downstream of Exon 10, which might affect the splicing process (Figure 2A and Supplementary Figure S1). Multiple alignment of SGLT2 protein showed that the three loci affected by missense variants (V116, S392 and V480) were evolutionarily conserved in all of the tested vertebrate species, indicating these amino acids substitutions might disrupt proper protein function (Figure 2B). We also analyzed the effects of these coding variants on the structure of SGLT2. Both p.V116M and p.Q445X variants showed palpable structural difference with the wild type SGLT2 (Supplementary Figures S2A,C). The p.V480F variant showed variable lengths of alpha folds at several positions compared to the wild type protein (Supplementary Figure S2D). In contrast, the structure of p.S392C variant seemed identical to the wild type SGLT2 (Supplementary Figure S2C). However, a more detailed analysis revealed the serine substitution by cysteine might disrupt the interaction between Ser392 and Ala73 (Supplementary Figure S3). Thus, all the coding variants may disrupt the proper structure of SGLT2. In accordance, these three missense variants were predicted to be “probably damaging” by PolyPhen-2 and SIFT softwares (10, 11) (Figure 2C). Taken together, these results suggest that all five identified SGLT2 variants are likely to be pathogenic.

## Discussion

In the present study, we utilized WES to probe into the genetic etiology of three Chinese children diagnosed with FRG and identified three novel compound heterozygous variants of SGLT2, which is the established causal gene for FRG. Routine urinary analysis showed glucose level above 3+ (mild to severe) in these patients. After following suggestions of increasing the uptake of carbohydrates and not overdoing exercise, these patients are showing improved symptoms, indicating favorable prognosis. Our bioinformatic analyses revealed that these variants were likely to impair the proper function of SGLT2. It will be important to perform functional experiments to verify whether these variants are truly disease-causing in future studies.

This study has two limitations. Since the probands had no family history of genetic kidney disease and their parents were normal, we did not perform urinary analysis on their parents. Besides, as the patients were in very young age (5, 1 and 4 years old, respectively), we found it difficult to collect 24-h urine and instead used qualitative urinary analysis for diagnosis.

In general, severe clinical consequences have not been observed in patients of FRG, which, therefore, is usually considered to be a benign condition (4, 12–14). Intriguingly, research on SGLT2 mutations-associated FRG have received a great of attention, as SGLT2 inhibitors, which usually show minimal side effects and include canagliflozin, dapagliflozin, and empagliflozin, have been clinically used to lower blood glucose level in patients with type 2 diabetes and are also showing promising potential for treatment of other diseases, such as cardiovascular disorders (15–17). Hence, identification of more pathogenic sites of SGLT2 is of clinical significance, which can provide novel insights into the mechanism of action of this transporter and thereby facilitate development of novel targeting inhibitors.

Molecular genetic studies have indicated an autosomal co-dominance with incomplete penetrance inheritance pattern for FRG (2–4). Patients with mild renal glucosuria usually result from heterozygous SGLT2 mutations, whereas patients with severe renal glucosuria are frequently caused by homozygous or compound heterozygous SGLT2 mutations (2, 3). Consistently, in our study, all three patients harbor compound heterozygous variants of SGLT2. Interestingly, the parents that are carriers of the variants, especially the p.Q445X truncating variant, appear normal, which are in line with previous reports that SGLT2 mutations may have variable expressivity among individuals (2–4). Our results, together with previous findings, suggest that other genes and environmental factors, such as diet, may have an impact on overall renal glucose transport.

## Conclusion

In this study, we describe three compound heterozygous variants of the SGLT2 gene in three Chinese pediatric FRG patients, which are predicted to be pathogenic and likely to account for the pathogenesis and progression of the disease. Among the total of five variants we have discovered, three ones are novel. Our findings expand the variant spectrum of SGLT2 in FRG and will aid in the development of novel SGLT2 inhibitors for treatment of other metabolic diseases.

## Data Availability

The datasets for this article are not publicly available due to concerns regarding participant/patient anonymity. Requests to access the datasets should be directed to the corresponding author.
