# Supplementary material for: Case report: Identification of three novel compound heterozygous SGLT2 variants in three Chinese pediatric patients with familial renal glucosuria
Source: Front Pediatr. 2022 Nov 28;10:996946. doi: 10.3389/fped.2022.996946 (PMC9742408; doi:10.3389/fped.2022.996946)
Supplement: Supplementary file 1 [file Datasheet1.docx]

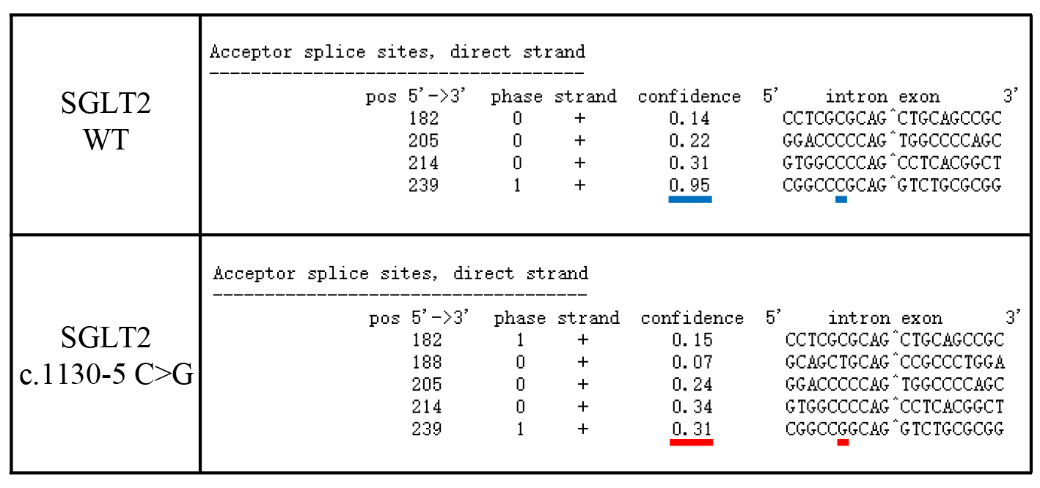


**Supplemental Figure 1.** The c.1130-5 C>G variant may interfere with splicing of SGLT2. The effect of c.1130-5 C>G variant on splicing was predicted by NetGene2 (https://services.healthtech.dtu.dk/service.php?NetGene2-2.42)[[1](#_ENREF_1)]. The C to G substitution caused the decrease of confidence from 0.95 to 0.31, indicating a possible loss of acceptor splice site.


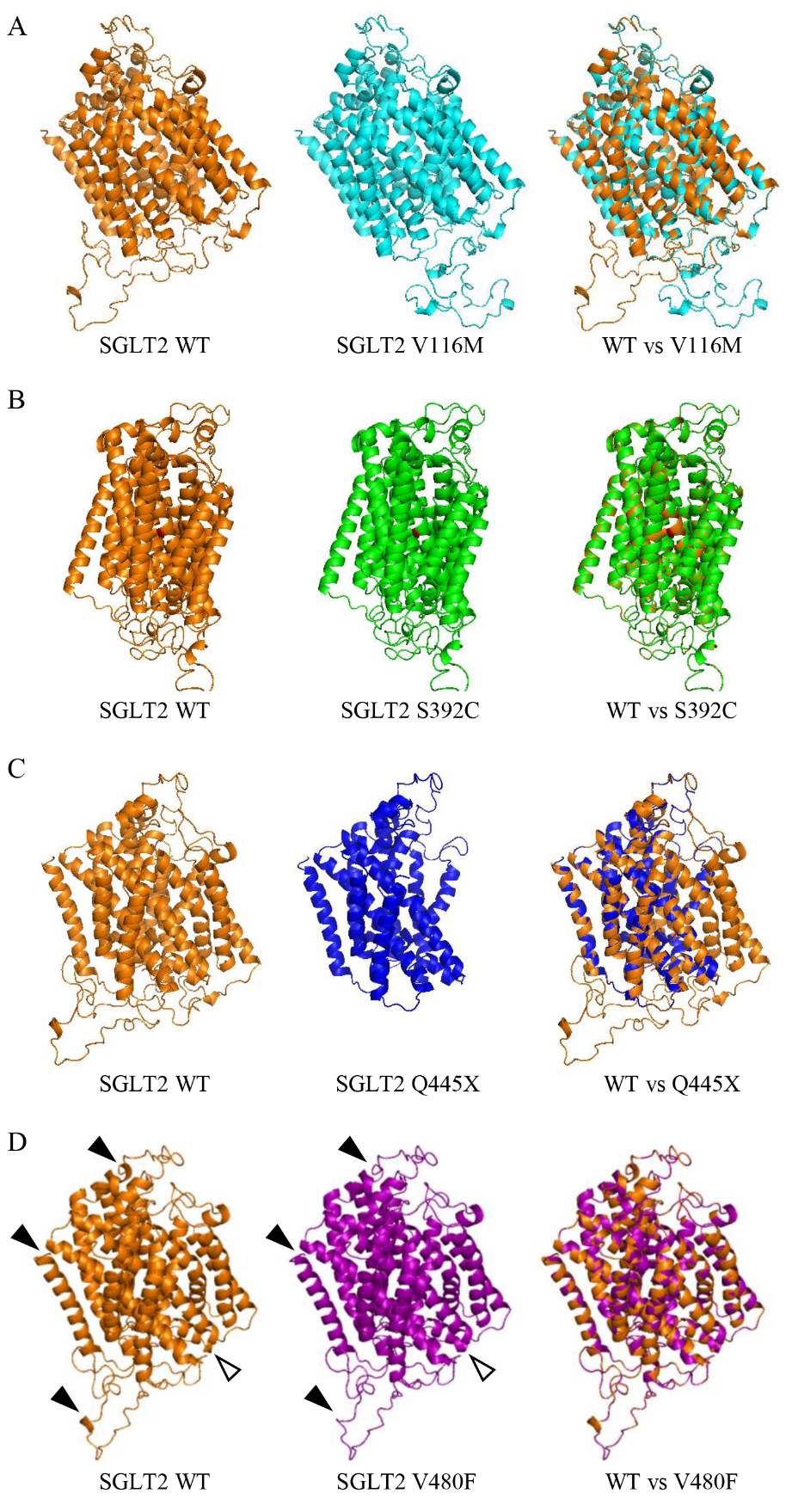


**Supplementary Figure 2**. Structural differences between wild type and mutant SGLT2 proteins. The structure of human SGLT2-MAP17 complex bound with empagliflozin (PDB coding: 7vsi.1.A) [[2](#_ENREF_2)] was used as the template to mimic the wild type and mutant SGLT2 by SWISS-MODEL (https://www.swissmodel.expasy.org/), and the difference of homology model were compared using PyMol [[3](#_ENREF_3)]. Arrows in D indicated different lengths of alpha folds.


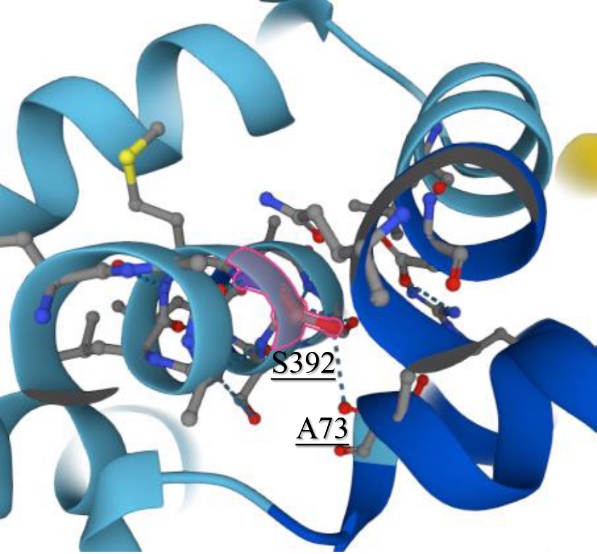


**Supplementary Figure 3**. The structure predicted by AlphaFold [[4](#_ENREF_4)] showed that there might be an interaction between Ser392 and Ala73, which was disrupted by serine substitution by cysteine.

| Clinical charateristics | Case1 | Case2 | Case3 |
| --- | --- | --- | --- |
| Age (years) | 5 | 1 | 4 |
| Gender | Male | Male | Female |
| Height (cm) | 114 | 79 | 100 |
| Weight (kg) | 21 | 12 | 15 |
| BMI | 16.16 | 19.22 | 15.00 |
| FPG (mmol/l) | 4.68 | 4.31 | 4.19 |
| HbA1C (%) | 3.90 | 3.72 | 4.78 |
| Blood insulin (µU/ml) | 5.16 | 4.73 | 3.43 |
| C-Peptide (ng/ml) | 1.15 | 1.29 | 0.86 |
| eGFR (ml/min/1.73m2) | 109.50 | 106.80 | 125.86 |
| Urine Glucose* | 3+ | 3+ | 3+ |
| Urine pH | 6.5 | 6.0 | 6.0 |
| SG | 1.020 | 1.031 | 1.015 |
| PRO (24-hour urinary protein quantity) | 18.6 | 12.18 | 25.1 |
| Urine amino acids | - | - | - |

**Supplemental Table 1.** Clinical characteristics of the patients. * Qualitative test for urine glucose was used for all the patients and their parents. +, 5.5mmol/l>urine glucose≥2.75mmol/l; 2+, 14mmol/l>urine glucose≥5.5mmol/l; 3+, 28mmol/l>urine glucose≥14mmol/l; 4+, urine glucose≥28mmol/l.

| Variants | Primer sequences |
| --- | --- |
| c.1333 C>T (p.Q445X) | Forward: TCCTCCCCAACGGATCAGCC |
|  | Reverse: ACGAAGAGCGCCAGCACGAA |
| c.1130-5 C>G | Forward: TGAAGCTCATGCCCAACGGTAA |
|  | Reverse: ACCGACACTACCACGATGAACA |
| c.346 G>A (p.V116M) | Forward: CAGGGATGAGGGCAAAGC |
|  | Reverse: CAGGAAAAGGGAGAGCACAGA |
| c.1438 G>T (p.V480F) | Forward: ATCGTGGTAGTGTCGGTGGC |
|  | Reverse: AGAAGAACAGCACAATGGCGA |
| c.1175 C>G (p.S392C) | Forward: CTGCTGGGAGGGGTCGTC |
|  | Reverse: CGCACCGTCCCACCAGC |

**Supplemental Table 2.** Primer sequences used for verification of variants by Sanger sequencing.

References:

1. Brunak, S., J. Engelbrecht, and S. Knudsen, *Prediction of human mRNA donor and acceptor sites from the DNA sequence.* Journal of molecular biology, 1991. **220**(1): p. 49-65.

2. Niu, Y., et al., *Structural basis of inhibition of the human SGLT2-MAP17 glucose transporter.* Nature, 2022. **601**(7892): p. 280-284.

3. Bramucci, E., et al., *PyMod: sequence similarity searches, multiple sequence-structure alignments, and homology modeling within PyMOL.* BMC bioinformatics, 2012. **13 Suppl 4**: p. S2.

4. Jumper, J., et al., *Highly accurate protein structure prediction with AlphaFold.* Nature, 2021. **596**(7873): p. 583-589.
